# Pulmonary aspergillus infection with abnormal imaging successfully treated with omalizumab: A case report

**DOI:** 10.1097/MD.0000000000033845

**Published:** 2023-06-16

**Authors:** Xing He, Zhenzhen Chang, Haiying Yan, Yang Weng

**Affiliations:** a Department of Pulmonary and Critical Care Medicine, Cheng Du Qing Cheng Mt. Hospital, Chongzhou City, Chengdu, China; b Department of Pulmonary and Critical Care Medicine, Sichuan Provincial People’s Hospital, University of Electronic Science and Technology of China, Chengdu, China; c School of Mathematics, Sichuan University, Chengdu, Sichuan, China.

**Keywords:** airway remodeling, case report, chest imaging, omalizumab, pulmonary aspergillus infection

## Abstract

Pulmonary aspergillosis is a pulmonary infectious disease that is clinically difficult to diagnose and treat. When the lower respiratory tract is invaded by Aspergillus, the clinical manifestations and imaging features vary among patients with different immune states. The use of antifungal drugs and glucocorticoids are important, but some patients do not respond satisfactorily to treatment.

**Case presentation:**

A 59-year-old female had a long history of asthma and poor symptom control, with long-term use of long-acting inhaled glucocorticoids combined with a long-acting β2 receptor agonists (ICS + LABA) (salmeterol fluticasone inhalation powder). The ground glass shadow, tree-in-bud sign, and bronchiectasis in the middle lobe of the right lung and the lower lobe of both lungs were first detected by chest CT over 5 years ago. Atelectasis in the middle lobe of the right lung was detected over 3 years ago. Over 2 years ago, the patient was hospitalized and a repeat chest CT showed persistent atelectasis in the middle lobe of the right lung, and more lesions in bilateral lower lungs than before. Aspergillus fumigatus was detected in alveolar lavage fluid and sputum pathogenic culture, which confirmed the diagnosis of pulmonary aspergillosis. After treatment with voriconazole and amphotericin B, the middle lobe of the right lung partially reopened, but the lesions in bilateral lower lungs persisted. After 21 weeks of treatment, the antifungal drugs were stopped because the patient refused to use oral/intravenous glucocorticoids, and omalizumab was finally chosen for treatment. After 1 month of treatment, the patient’s clinical symptoms began to ease. After 1 year of treatment, imaging reexamination of lung showed that the lesions were completely cleared, accompanied by significant improvement in nutritional status and airway function.

**Conclusions:**

We reported the case of a patient with pulmonary Aspergillus infection who was treated with omalizumab and showed significant improvement in clinical symptoms and imaging abnormalities, which provides a new option for patients with pulmonary Aspergillus infection who show unsatisfactory response with first-line drugs.

## 1. Introduction

Pulmonary aspergillosis is a general term for lung diseases caused by Aspergillus infection, mainly including invasive pulmonary aspergillosis (IPA), allergic bronchopulmonary aspergillosis (ABPA), and chronic pulmonary aspergillosis (CPA), with Aspergillus fumigatus as the most common pathogen.^[1]^ The clinical features and imaging manifestations of Aspergillus infection vary according to individual immune status. If the pathogenic serum immunology and culture results are negative, it may pose a challenge for the classification and diagnosis of the disease. A clear classification is critical for guiding the use of medication. For example, IPA and CPA treatments are focused on triazole drugs, ABPA also requires a combination of oral/intravenous glucocorticoids due to the allergic reaction of the pulmonary parenchyma induced by the inhalation of Aspergillus at the end of the respiratory tract. In the real world, patients with pulmonary Aspergillus infection who do not meet the classification criteria are judged as suspected cases by clinicians, and the treatment strategy for such patients is very passive.^[2]^ Such patients not only face the adverse effects caused by therapeutic drugs, such as visual impairment and liver damage caused by voriconazole but also about 1 in 4 patients do not respond to therapeutic drugs.^[3]^ Therefore, how to choose a safe and effective treatment plan needs attention.

Omalizumab is a novel monoclonal antibody targeting IgE, which blocks IgE-associated immune pathways by binding to IgE. It is mainly used in the treatment of allergic diseases. Past studies have found that omalizumab is beneficial in ABPA treatment, including improvement in clinical symptoms and lung function, and reduction in hormone use.^[4]^ We reported a patient with asthma with pulmonary Aspergillosis infection who was unresponsive to multiple first-line therapeutic agents and was treated with omalizumab, which resulted in effective improvement of clinical symptoms as well as complete elimination of pulmonary imaging abnormalities.

## 2. Case presentation

**Figure 1.**
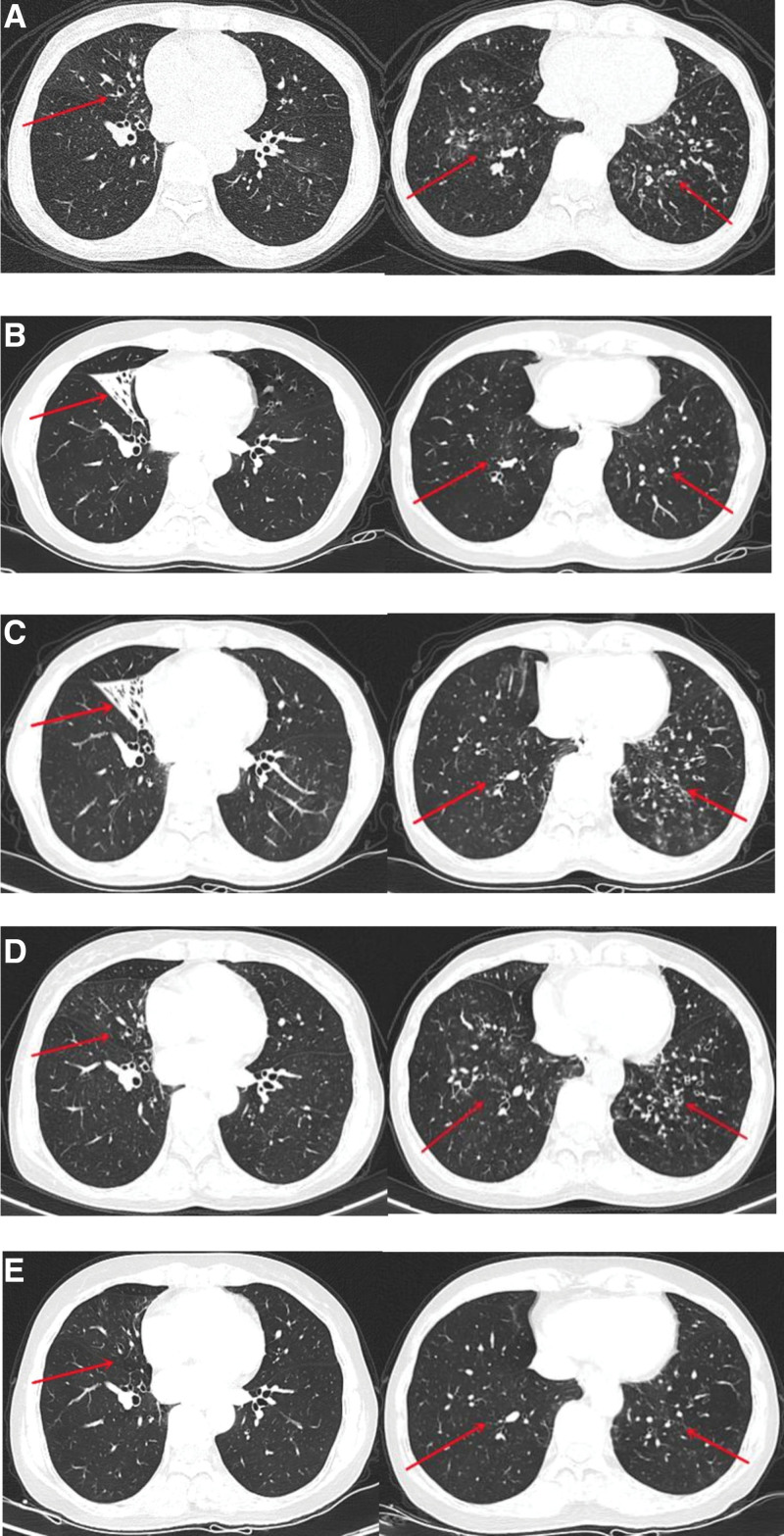
Imaging characteristics of chest lesions of our patient before and after treatment. (A–E): (A) In February 2017, the imaging abnormality of lung was first found. (B) In February 2019, atelectasis was first found in the middle lobe of the right lung with reduced lesions in bilateral lower lungs (ground glass shadow, tree-bud sign, mucus plug, bronchiectasis) compared to before. (C) Pulmonary imaging prior to antifungal treatment with persistent atelectasis in the middle lobe of the right lung and increased lesions in bilateral lower lungs in April 2020. (D) Lung imaging reexamination after antifungal treatment showed improvement of atelectasis in the middle lobe of the right lung, with no significant change in bilateral lower lung lesions in October 2020. (E) In November 2021, 1 year after treatment with omalizumab, the reexamination showed that atelectasis in the middle lobe of the right lung and the lesions of both lower lungs were eliminated (the red arrow marked the main positions of lesions before and after treatment).

A 56-year-old female patient complained of repeated coughing and wheezing for over 10 years, with allergic rhinitis, allergy to milk, seafood, and many other substances. Over 10 years ago, she developed recurrent cough, wheezing and chest tightness, which was significant in summer and winter, and was subsequently diagnosed as asthma at the local hospital. The patient was started on ICS + LABA (salmeterol fluticasone inhalation powder 50 µg/250 µg) twice daily, and her symptoms were unsatisfactorily controlled. Five years ago, a ground glass shadow, and tree-bud sign in the middle lobe of right lung and lower lobe of both lungs with bronchiectasis were first detected by chest CT. Three years ago, chest CT reexamination showed persistence of the lesions in bilateral lower lungs, along with atelectasis of middle lobe of right lung. Two years ago, the symptoms of cough, expectoration and dyspnea persisted without relief. The outpatient examination showed that FeNO was 41 ppb, and then she was admitted to the hospital, with BMI 16.7, normal serum neutrophil and eosinophil count, negative GM, serum total IgE 152 IU/mL, Aspergillus fumigatus specific IgE 0.28 IU/mL. Bronchoscopy showed more airway secretions, but no airway obstruction was found. Aspergillus fumigatus was detected in bronchoalveolar lavage fluid and sputum pathogenic culture, and the final diagnosis was pulmonary Aspergillosis with uncertain classification (Table 1). Therefore, oral/intravenous voriconazole was given for 16 weeks, and the dose of inhaled ICS + LABA was increased (salmeterol fluticasone inhalation powder 50 µg/500 µg). The middle lobe atelectasis of the right lung had improved on repeat CT, but the lesions of bilateral lower lungs had not changed. Then, combined aerosol inhalation of amphotericin B 25 mg twice a day was given for 5 weeks, after which the asthma symptoms and lung lesions of the patient remained unchanged. Considering the past allergic rhinitis with increased FeNO and serum IgE levels, allergic airway inflammation was suspected. However, the patient was concerned about adverse drug reactions and refused oral/intravenous glucocorticoids, so we finally decided to use inhaled ICS + LABA (salmeterol fluticasone inhalation powder 50 µg/500 µg) combined with omalizumab 300 mg once every 4 weeks. After 1 month of treatment, the patient’s clinical symptoms began to ease, and were gradually controlled and stabilized. After 1 year, the reexamination showed that the pulmonary imaging lesions had normalized (Fig. 1A–E), the nutritional status (BMI 20.1) was improved, FeNO was lower than before (30 ppb), and small airway function was improved.

**Table 1:** Clinical features of the reported patients, classification and exclusion criteria of pulmonary aspergillosis infection*.

Patient characteristics	Type	Recommended guide source	Troubleshooting causes
1. History of allergic rhinitis and asthma no tumor, no oral/intravenous hormones and immunosuppressants were used. 2. Repeated cough, expectoration and dyspnea for more than 10 yr. 3. Serum total IgE level increased. The number of neutrophils in blood is normal. Serum GM (−) and serum aspergillus fumigatus specific IgE (−) 4. Chest CT: atelectasis of middle lobe of right lung, ground glass shadow, tree-bud sign. 5. Etiology: bronchoalveolar lavage fluid and sputum culture: aspergillus fumigatus (+) 6. Voriconazole combined with amphotericin has poor symptom control and imaging improvement.	IPA	De Pauw, Ben et al.^[5]^	Sputum culture and BALF results cannot be used as the basis for diagnosis. Serum GM (−) does not match the low immune host.
	ABPA	Agarwal, R et al.^[6]^	Does not meet the mandatory standards: serum total IgE and aspergillus fumigatus specific IgE do not meet the standards.
	CPA	Denning, David W et al.^[7]^	Imaging does not have: chronic imaging manifestations such as fungus ball, cavity and fibrosis. After treatment, pulmonary lesions were cleared.

ABPA = allergic bronchopulmonary aspergillosis, CPA = chronic pulmonary aspergillosis, IPA = invasive pulmonary aspergillosis.

* Judgment was made according to the patient’s clinical features, combined with the expert consensus or clinical guidelines of pulmonary aspergillosis.

## 3. Discussion

It is difficult to identify pulmonary aspergillosis infection and the accompanying allergic state. When Aspergillus invades the lower respiratory tract, the body responses differ according to the individual autoimmune status. In immunocompromised conditions, IPA is easily manifested, such as in people receiving chemotherapy or using immunosuppressants. Another manifestation is CPA, which requires patients to have chronic pulmonary or systemic symptoms lasting for at least 3 months, accompanied by features such as Aspergillus nodules, Aspergillus globules, and cavities on chest imaging. ABPA is an allergic reaction after pulmonary infection with Aspergillus, manifested by repeated asthma attacks and chest images showing signs of bronchodilation, mucus embolism and pulmonary nodules. The patient did not fit either of the diagnoses, but we still administered antifungal drugs for 21 weeks according to the results of pathogenic culture. However, the clinical symptoms and imaging abnormalities did not improve after treatment. We speculated that this may be an allergic reaction caused by Aspergillus infection, resulting in indistinguishability from infectious inflammation. Right middle lobe atelectasis, ground glass shadow of bilateral lower lungs, tree-bud sign and bronchiectasis were the important features of this patient. Fungal infection, bronchial asthma and ABPA can present with right middle lobe atelectasis.^[8]^ The patient also had allergic rhinitis, asthma, high levels of serum IgE, etc. These allergic reaction-related features supported change of the treatment plan and we planned to start oral/glucocorticoids, which was rejected by the patient for fear of adverse effects on immune and endocrine metabolism. Therefore, we finally started omalizumab treatment with inhaled ICS + LABA according to the recommended protocol of 2020 GINA guidelines. After 1 month of treatment, the symptoms were relieved, and subsequent review revealed complete clearance of pulmonary CT abnormalities. These findings indicated that when pulmonary Aspergillus infection is treated with omalizumab, it not only provides symptom control but also the advantage of clearing pulmonary imaging abnormalities after Aspergillus infection, thereby avoiding the use of oral/intravenous glucocorticoids.

Immunological evidence also supports our choice of drug with Aspergillus mediating the host-pathogen immune response through spores and hyphae.^[9]^ Previous studies have shown that Aspergillus can promote lung epithelial cells to release more IL-13 and IL-17.^[10]^ IL-17 can exacerbate airway inflammation, increase airway hyper-responsiveness and reduce the response of glucocorticoid therapy,^[11]^ leading to poor effect of glucocorticoid and persistent inflammatory state. However, IL-13 can further induce IgE production by B lymphocytes following release from Th2 cells, and IgE binding to mast cells causes a more persistent allergic response, further enhancing the immune effects of the associated inflammatory cells as IgE concentration increase in the circulation or locally and continuing to act even after clearance of Aspergillus. The use of omalizumab can inhibit IgE binding to inflammatory cell surface receptors while reducing the level of IL-13 in seru,^[12]^ directly blocking inflammatory cells (mast cells, basophils) from exerting their effects. A recent study found a reduction in serum IL-17 levels after 38 weeks of omalizumab use.^[13]^ This means that omalizumab can block IgE effector pathways and key cytokine levels in multiple ways, without relying on glucocorticoid involvement, to suppress the allergic state following Aspergillus infection.

In chronic airway diseases, the stimulation of the airway by a persistent inflammatory state causes bronchial wall thickening, airway stenosis, and finally leads to airway remodeling. Studies have shown that IgE-related pathways and cytokines such as IL-4 and IL-13 are involved in airway remodeling.^[14]^ Therefore, targeting IgE and IL-4/IL-13 axis can potentially improve airway remodeling. The use of omalizumab can inhibit IgE-related pathways, reducing IL-4 and IL-13 levels, and exerting its ability to improve airway remodeling by controlling airway inflammation and reducing collagen deposition.^[15]^Our patient was treated with omalizumab, which cleared abnormal lung imaging chronic lesions, indicating that omalizumab has the ability to eliminate airway inflammation following pulmonary Aspergillosis infection and reverse airway remodeling.

## 4. Conclusions

This report shows that omalizumab can control asthma symptoms in patients with pulmonary Aspergillosis, as well as simultaneously eliminate the pulmonary imaging abnormalities in patients without relying on oral/intravenous glucocorticoid administration, which provides an alternative treatment option for patients with pulmonary Aspergillosis, especially those who may have allergic reactions following Aspergillosis infection.

## Author contributions

**Conceptualization:** Xing He, Haiying Yan.

**Data curation:** Zhenzhen Chang, Haiying Yan, Yang Weng.

**Formal analysis:** Zhenzhen Chang, Haiying Yan.

**Methodology:** Xing He.

**Project administration:** Xing He.

**Resources:** Yang Weng.

**Writing – original draft:** Xing He, Haiying Yan.

**Writing – review & editing:** Xing He, Haiying Yan.
